# Quasi-One-Dimensional Linarite-Type PbCu(SeO_4_)(OH)_2_ with Competing Nearest-Neighbor and Next-Nearest-Neighbor Intrachain Exchange Interactions

**DOI:** 10.3390/ma15217860

**Published:** 2022-11-07

**Authors:** Maria Markina, Konstantin Zakharov, Yevgeniy Ovchenkov, Grigoriy Pashkov, Konstantin Lyssenko, Petr Berdonosov, Zlata Pchelkina, Alexander Vasiliev

**Affiliations:** 1 Lomonosov Moscow State University, Moscow 119991, Russia; 2 National University of Science and Technology “MISiS”, Moscow 119049, Russia; 3 Ural Federal University, Ekaterinburg 620002, Russia; 4 Institute of Metal Physics, RAS, Ekaterinburg 620108, Russia

**Keywords:** linarite, low-dimensional magnetism, magnetic phase diagram

## Abstract

PbCu(SeO_4_)(OH)_2_, the selenate sibling of the mineral linarite, was synthesized hydrothermally, investigated in measurements of magnetization *M*, specific heat *C_p_* and dielectric permittivity *ε*, and analyzed within density functional theory formalism. This quasi-one-dimensional compound evidences formation of a short-range correlation regime at T* ~ 8 K and experiences a long-range magnetic order at *T_N_* = 4.3 K. A magnetization saturation of approximately 1 *µ_B_* is reached at *µ_0_H_flip_* ~ 16 T preceded by a jump at *µ_0_H_flop_* = 2.4 T. Additionally, there are multiple indicators of the formation of an additional electrically active phase above the Neel temperature, which suggests that PbCu(SeO_4_)(OH)_2_ is a multiferroic system.

## 1. Introduction

Quasi-one-dimensional uniform spin systems with competing intrachain exchange interactions attract attention by formation of exotic ground states and the presence of quantum critical points between these states due to frustration of the nearest-neighbor exchange interaction *J_nn_* by the next-nearest-neighbor exchange interaction *J_nnn_*. Depending on the signs and values of these interactions, various spin arrangements are possible at low temperatures [1]. When *J_nnn_* is antiferromagnetic, *J_nnn_* > 0, the chain is frustrated independent of the sign on *J_nn_* [2]. It is predicted, but never observed experimentally, that a spin gap opens at *J_nnn_*/*J_nn_* = *α* > *α_c_* = 0.241 [3]. At *α* = 0.5, the Majumdar–Ghosh ground state is expected as a superposition of spin singlets [4]. It was found, however, that the copper chromate, CuCrO_4_, supposed to be the best realization of this model with *J_nn_* = 54 K and *J_nnn_* = 27 K orders antiferromagnetically at *T_N_* = 8.25 K due to the non-negligible interchain interactions [5]. If *J_nn_* is negative (*J_nn_* < 0), the ferromagnetic order within the chain should established in the range −0.25 < *α* ≤ 0. At *α* = −0.25, the system undergoes quantum phase transition to an incommensurate spin helix state [6]. Among numerous species of this type, there are many cuprates, among them LiCuVO_4_ [7], LiCu_2_O_2_ [8], Li_2_CuZrO_4_ [9], and LiCuSbO_4_ [10]. Some of these compounds evidence the multiferroic properties.

Over the last decade, there was a surge of interest in the mineral linarite PbCu(SO_4_)(OH)_2_, which hosts the well separated spin-1/2 uniform chains of Cu(OH)_2_ units. The ferromagnetic nearest-neighbor intrachain exchange interaction in this system is frustrated by the next-nearest-neighbor intrachain antiferromagnetic exchange interaction [11]. This material experiences a long-range magnetic order at *T_N_* = 2.8 K preceded by a phase, which was identified as an incommensurate longitudinal spin density wave state with a wavevector dependent on both temperature and magnetic field [12,13,14,15,16,17]. Below *T_N_*, the linarite evidences sequence of spin-flop and spin-flip transitions. Further, at the lowest temperatures and in near the Neel temperature, two more hysteretic phases were found, which suggests the coexistence of commensurate and incommensurate circular spin orders [18,19,20,21]. Magnetic ordering in PbCu(SO_4_)(OH)_2_ is accompanied by anomalies in dielectric permittivity and electrical polarization, which makes this material type-II multiferroic [18,22,23].

Here, we present the newly synthesized selenium counterpart, PbCu(SeO_4_)(OH)_2_, of the mineral linarite. It was characterized in measurements of magnetization *M* in both static and pulsed magnetic fields, dielectric permittivity *ε* and specific heat *C_p_* and analyzed within density functional theory formalism.

## 2. Synthesis and the Crystal Structure

The selenate sibling of linarite, PbCu(SeO_4_)(OH)_2_, was prepared under hydrothermal conditions. CuCl_2_·2H_2_O, PbO (Reachim) and H_2_SeO_4_ (80%) (Vekton, Penfield, NY, USA) were used for the synthesis. PbSeO_4_ was prepared in a 25 mL autoclave loaded with 1.1221 g of PbO and 1 mL of H_2_SeO_4_ and kept at 200 °C for a week. The white precipitate was filtered from the solution washed by deionized water and dried on air. The purity of the sample was confirmed by powder XRD on a STOE STADI-P diffractometer (Cu K_α1_ radiation) and the ICDD PDF 2 database was used as a reference. The X-ray pattern was in agreement with the 00-015-0375 PDF 2 dataset of PDF2. Prepared lead selenate was used for PbCu(SeO_4_)(OH)_2_ preparation.

Prepared PbSeO_4_ 0.8000 g (2.285 mmol) and CuCl2·2H_2_O 0.5899 g (3.459 mmol) were mixed with 5 mL of 5% NaOH water solution in a 25 mL autoclave stirred by a glass rod and heated in a furnace for 4 days at 150 °C. As a result, a dark blue precipitate was formed. After filtering under vacuum and washing by distilled water, the precipitate was dried. The examination by an optical microscope shows the formation of small blue crystals. Powder XRD shows the analogy with PbCu(SO_4_)(OH)_2_. The pattern was indexed assuming the existence of a *P2_1_/m* space group with cell constants *a* = 4.7721(10) Å, *b* = 5.7633(12) Å, *c* = 9.869(3) Å, *β* = 102.616(16)°, cell volume 264.87(16) Å^3^, and Figure of Merit F(30) = 38.5. The conclusion on the linarite-type phase formation was made based on these data.

The single crystal of PbCu(SeO_4_)(OH)_2_ was investigated on a Bruker D8 QUEST single-crystal X-ray diffractometer equipped with a PHOTON II detector, a charge-integrating pixel array detector (CPAD), a lsaterally graded multilayer (Goebel, Bad Staffelstein, Germany) mirror and a microfocus Mo-target X-ray tube (λ = 0.73071 Å). A frame width of 0.5° and exposure time of 2 s/frame were employed for data collection. Data reduction and integration were performed with the Bruker software package SAINT (Version 8.40B) [24]. The data were corrected for Lorentz and polarization effects.

Absorption correction was performed using a multiscan routine as implemented in SADABS (Version 2016/2) [25]. Atomic positions were located using direct methods and refined using a combination of Fourier synthesis and least-squares refinement in isotropic and anisotropic approximation. All calculations were carried using SHELXTL PLUS software [26]. The crystallographic parameters and final residuals for the single-crystal XRD experiments are given in Table 1. A summary of the crystallographic data for the single-crystal experiments is available from CCDC, ref. number 2172183.

**Figure 1:**
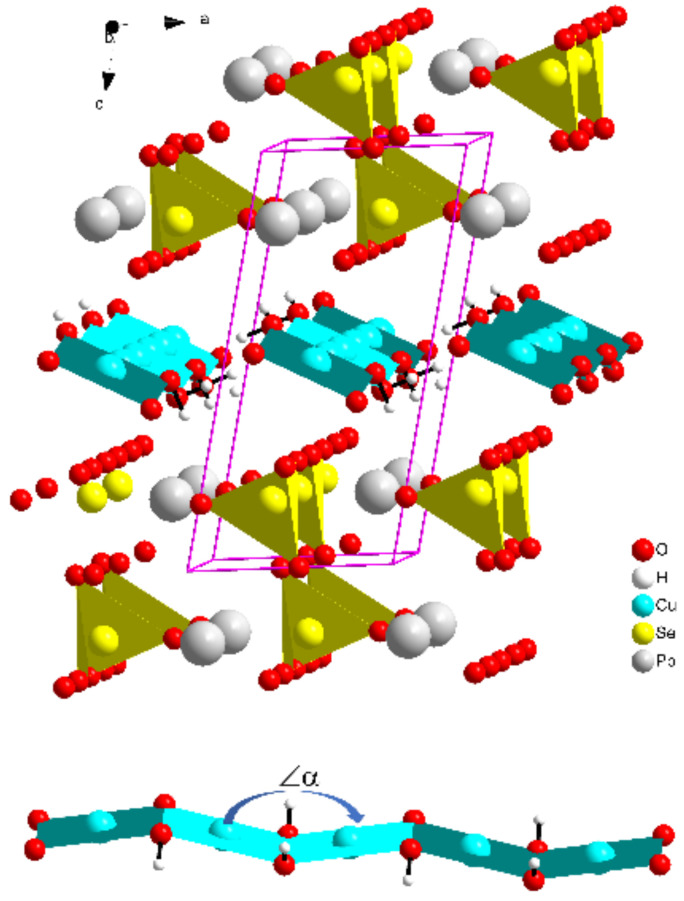
The crystal structure of PbCu(SeO_4_)(OH)_2_. CuO_4_ squares and SeO_4_ groups are shown. The chain of CuO_4_ squares is shown at the low part of the figure.

The experiment shows that the unit cell of PbCu(SeO_4_)(OH)_2_ contains one Pb, one Cu and one Se atom. Among five oxygen atoms, two belong to OH groups. According to the literature data [27,28], the mineral linarite crystallizes in a monoclinic space group *P2_1_/m* (*a* = 9.682(2), *b* = 5.646(1), *c* = 4.683(6) Å, and *β* = 102.66(1)°). The buckled chains of edge-sharing CuO_4_ squares are running in the crystal structure parallel to the [010] direction, as shown in Figure 1. These squares are parts of the strongly Jahn–Teller-distorted CuO_6_ bipyramids with Cu-O difference from 1.93–1.98 to 2.54 Å. The layers of hydrogen-bonded Cu-OH chains are connected by PbO_8_-distorted polyhedra and SO_4_ tetrahedra.

The nearest-neighbor surrounding of the Cu atom in PbCu(SeO_4_)(OH)_2_ is formed by the O4 and O5 oxygen atoms from hydroxyl groups with of distances 1.93–1.99 Å in the square planar CuO_4_ group. According to the Jahn–Teller effect, an additional two O2 atoms may be included in the copper atom surrounding at a distance of 2.48 Å, as presented in Table 2. CuO_4_ squares share their O4-O5 edges and form zig-zag chains along the [010] direction. The average <Cu-O> distance of CuO_4+2_ bipyramids in PbCu(SeO_4_)(OH)_2_ is 2.136 Å, being close to that reported for linarite, 2.148 Å [28]. The angle *α* between the two nearest CuO_4_ squares in the selenium compound, 159.02°, is larger than that in linarite, 154.70° [28].

Corresponding to the data reported in [27,28], the Pb atom in linarite lays within an eight-vertex oxygen polyhedron and is shifted from the center of the polyhedron, manifesting electron lone-pair activity. In the case of PbCu(SeO_4_)(OH)_2_, there are seven nearest oxygen atoms for the Pb atom. The next oxygen atom lays on the distance 3.2 Å. The selenium atom in the selenate SeO_4_^2−^ group lays in a tetrahedron with Se-O distances in the usual range, 1.63–1.65 Å.

## 3. Physical Properties

Magnetization *M* and specific heat *C_p_* in PbCu(SeO_4_)(OH)_2_ in the range 2–330 K under a magnetic field *µ_0_H* up to 9 T were studied using relevant options of “Quantum Design” Physical Properties Measurements System PPMS-9T. Measurements in a pulsed magnetic field up to 32 T with pulse duration of approximately 10 msec were provided by a homemade capacitance bank discharge setup. Dielectric permittivity *ε* was measured on the platform of “Quantum Design” Magnetic Properties Measurements System MPMS-7T by means of an Andeen–Hagerling bridge AH2700 in the frequency range *f* = 10^2^–10^4^ Hz on the thin pressed pellets covered with a silver paste.

**Figure 2:**
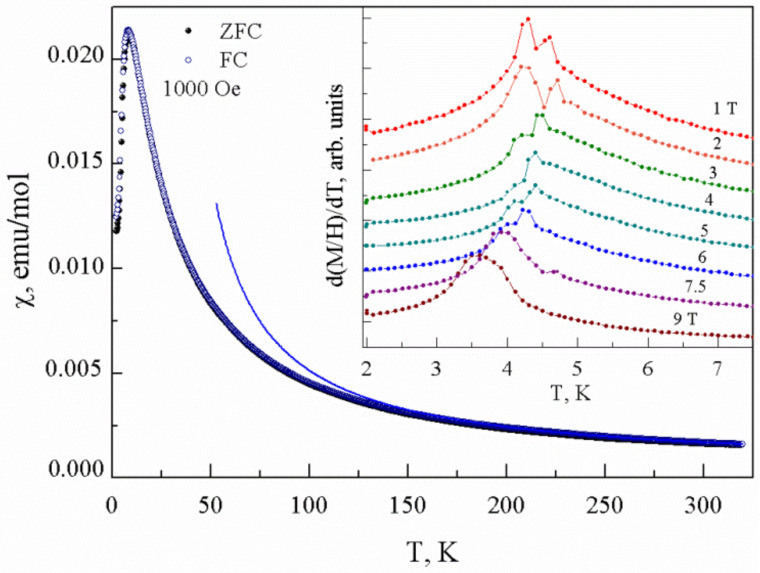
Temperature dependences of magnetic susceptibility *χ* in PbCu(SeO_4_)(OH)_2_ taken at *µ_0_H* = 0.1 T in both FC and ZFC regimes. Inset: *dχ*/*dT* curves taken at various magnetic fields.

The temperature dependences of magnetic susceptibility *χ* = *M/H* taken at *µ_0_H* = 0.1 T in both the field-cooled (FC) and zero-field-cooled (ZFC) regimes are shown in Figure 2. No difference between these curves were detected within the experimental resolution, which signals the absence of pronounced impurities. At elevated temperatures, the *χ*(*T*) can be described by the Curie–Weiss law

(1) $$\chi =\chi_{0}+\frac{C}{T-\Theta }$$

with the temperature-independent term *χ*_0_ = 4.1 × 10^−4^ emu/mol, the Curie constant *C* = 0.355 emu K/mol and the Weiss temperature *Θ* = 25 K. In accordance with the ratio 8*C* = *µ_eff_*^2^, the effective magnetic moment *µ_eff_* = 1.68 *µ_B_*, which is slightly lower than the expected value, 1.73 *µ_B_*, for *g*—factor *g* = 2. The reduction in the effective magnetic moment *µ_eff_* is a standard signature of low-dimensional magnetic systems. On lowering the temperature, the experimental data deviate downward from the extrapolation of the Curie–Weiss law, which points to the presence of antiferromagnetic interactions in the system. At approximately T* ~ 8 K, the *χ*(*T*) curve evidences a hump followed by the establishment of the long-range magnetic order at *T_N_* = 4.3 K. The Neel temperature is marked by a peak in the temperature dependence of the derivative *dχ*/*dT*, as shown in the inset to Figure 2. This feature, however, is split and broadens at higher magnetic fields.

**Figure 3:**
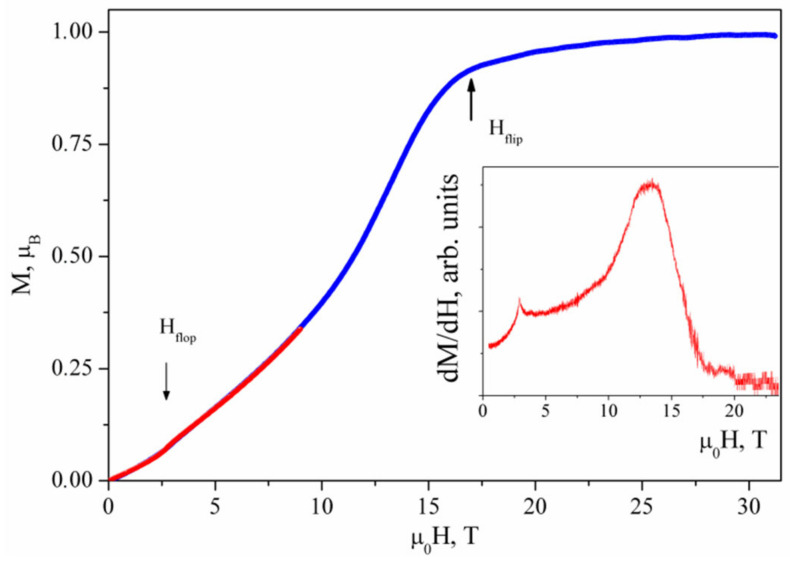
Field dependence of magnetization *M* in PbCu(SeO_4_)(OH)_2_ in a pulsed magnetic field at *T* = 2.4 K normalized by measurements in the static magnetic field. Inset: *dM*/*dH* curve.

The field dependence of magnetization *M* in a pulsed magnetic field taken at 2.4 K is shown in Figure 3. This curve is normalized by the *M*(*H*) curve taken in the static magnetic field at the same temperature. The saturation at the level of 1 *µ_B_* is reached at *µ_0_H_flip_* ~ 16 T. It is preceded by a jump at *µ_0_H_flop_* = 2.4 T. The anomalies at spin-flop and spin-flip transitions are pronounced in the field dependence of derivative dM/dH, as shown in the inset to Figure 3. An additional small anomaly can be identified at *µ_0_H* ~ 19 T.

**Figure 4:**
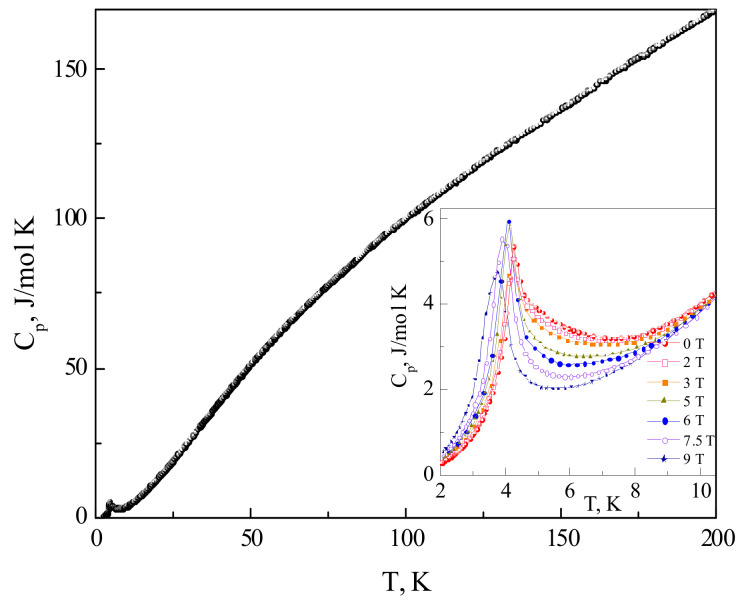
Temperature dependence of specific heat *C_p_* in PbCu(SeO_4_)(OH)_2_. Inset: *C_p_*(*T*) curves taken at various magnetic fields.

The temperature dependence of specific heat *C_p_* in PbCu(SeO_4_)(OH)_2_ is shown in Figure 4. The *C_p_*(*T*) curve evidences the presence of a sharp λ-type anomaly at *T_N_* = 4.3 K characteristic to the second-order phase transition. At 200 K, which is the upper limit of measurements, the value of specific heat is still far from the thermodynamic limit, 3*Rn* = 275 J/mol K, where *R* is the universal gas constant and *n* is the number of atoms per formula unit. Under an external magnetic field, this anomaly shifts to lower temperatures, as shown in the inset to Figure 4. In the zero field, the peak is preceded by a pre-transition region, which is strongly suppressed by the magnetic field. It makes the anomaly in strong fields more pronounced, i.e., the Δ*C_p_* at the transition point increases.

**Figure 5:**
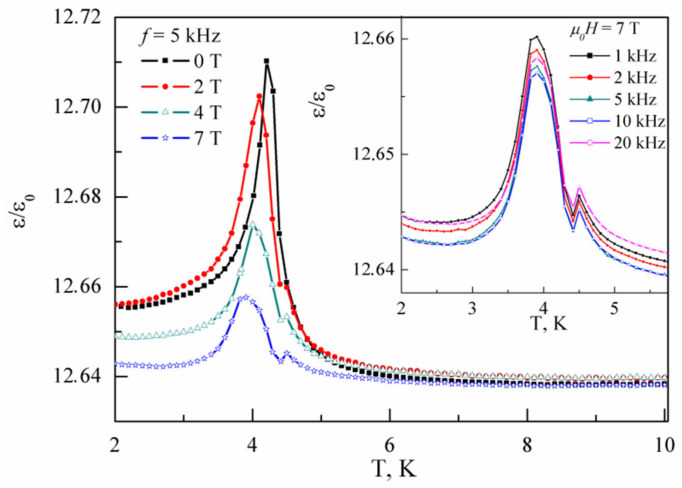
Temperature dependences of dielectric permittivity *ε/ε_0_* in PbCu(SeO_4_)(OH)_2_ taken at *f* = 5 kHz in various magnetic fields. Inset: temperature dependences of dielectric permittivity taken at *µ_0_H* = 7 T for various frequencies.

The temperature dependences of dielectric permittivity *ε/ε_0_* in PbCu(SeO_4_)(OH)_2_ taken at *f* = 5 kHz in various magnetic fields are shown in Figure 5. These curves evidence a sharp peak at the Neel temperature *T_N_* = 4.3 K, which shifts to lower temperatures under an external magnetic field. Further, an additional anomaly seen only in the presence of magnetic field arose at 4.5 K. In the range 1–20 kHz, the positions of these anomalies are not frequency dependent, as shown in the inset in Figure 5. The appearance of an additional anomaly above Neel temperature indicates that the electrically active phase forms in PbCu(SeO_4_)(OH)_2_ prior to the long-range magnetic order, similar to observations in the mineral linarite [22,23].

**Figure 6:**
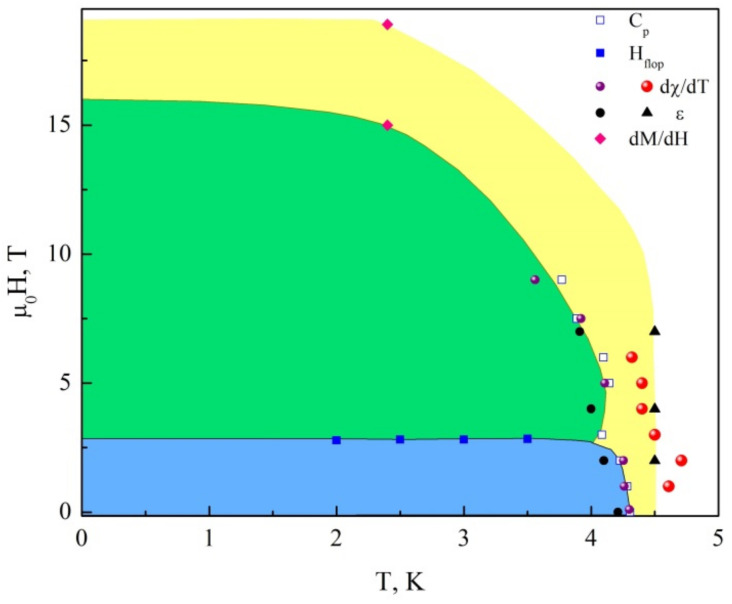
Magnetic phase diagram in PbCu(SeO_4_)(OH)_2_ as derived from measurements of magnetization *M*, specific heat *C_p_* and dielectric permittivity *ε*.

The combination of experimental data obtained in magnetization *M*, specific heat *C_p_* and dielectric permittivity *ε* measurements enables establishing the magnetic phase diagram in PbCu(SeO_4_)(OH)_2_, as shown in Figure 6. The spin-flop boundary subdivides a magnetically ordered state into two phases prior to saturation magnetization. There are also multiple signatures of an additional phase preceding the long-range antiferromagnetic order similar to observations in the mineral linarite [22,23].

## 4. Density Functional Calculations

The exchange interaction parameters within Cu^2+^ chains in PbCu(SeO_4_)(OH)_2_ and, for comparison, in PbCu(SO_4_)(OH)_2_ were obtained by calculating the total energy difference of various magnetic configurations and mapping on the spin model with the Hamiltonian

(2) $$H=J_{1}\sum S_{i}S_{i+1}+J_{2}\sum S_{i}S_{i+2}$$

The ab initio band structure calculations were carried out within the framework of the density functional theory (DFT) as implemented in VASP [29,30,31]. The generalized gradient approximation (GGA) [32] for the exchange-correlation functional was used. Conventional DFT calculations underestimate the effect of strong Coulomb correlations important for correct description of the magnetic ground state of transition metal oxides. Therefore, we perform GGA+U [33] calculations taking into account strong Coulomb correlations on Cu sites. We use the values of the Hund coupling *J_H_* = 1 eV and the on-site Hubbard repulsion parameter, *U* = 7 eV, as in [12], and also *U* = 9 eV, which is close to the *U* = 9.5 eV obtained from the linear-response method [34] in [35] for the Cu_2_GeO_4_ compound. The cutoff energy for plane waves was chosen to be 500 eV. The integration over the Brillouin zone was performed using the 4 × 4 × 4 k-mesh. In order to calculate exchange interaction parameters both along the chain and between neighboring chains, the supercells consisting of 8 formula units (f.u.) were used. The obtained exchange interaction parameters, *J_i_*, are listed in Table 3.

## 5. Discussion

Linarite, PbCu(SO_4_)(OH)_2_, is a prototype half-integer spin chain material with competing ferromagnetic nearest-neighbor and antiferromagnetic next-nearest-neighbor exchange interactions. On the basis of magnetic susceptibility and specific-heat data, the predominant ferromagnetic nearest-neighbor (NN) exchange interaction *J*_1_ = −30 K and weaker antiferromagnetic next-nearest-neighbor (NNN) exchange interaction *J*_2_ = 15 K (*α* = 0.5) were established [11]. This strong-coupling scenario was, however, in contrast to the values *J*_1_ = −13 K and *J*_2_ = 21 K (*α* = 1.6) obtained from the fit of the susceptibility data using a high-temperature expansion up to the fourth order at 50 < *T* < 350 K [22]. In order to resolve a controversy, the comprehensive experimental and theoretical study of PbCu(SO_4_)(OH)_2_ was undertaken. Measurements of magnetic susceptibility and saturation magnetization as well as electron spin resonance and nuclear magnetic resonance were performed [12]. The theoretical analysis within the density matrix renormalization group, the hard-core boson technique as well as ab initio calculations using local spin-polarized density approximation (LSDA+U) yield values of the main couplings of *J*_1_ = −133 K and *J*_2_ = 42 K [12]. The value of *J*_1_ = −118 K obtained within VASP (Table 3) for *U* = 7 eV is in agreement with that calculated within FPLO (the Full-Potential Local-Orbital electronic structure code [36]) in [12], while *J*_2_ = 72 K is much larger. We have to point out that while the reproducibility of results obtained by different DFT codes is well established (see for example [37]), the implementation of *U* correction, on the contrary, could be different depending on the basis set used in various codes. For example, the dramatic difference in the values of exchange interactions calculated within the FPLO and VASP methods were previously reported for the Cu_2_GeO_4_ compound [35]. In this compound, Cu and O form infinite chains of edge-sharing plaquettes linked into a 3D network via the non-magnetic GeO_4_ tetrahedra. DFT calculations were performed in different codes that delivered largely consistent results for *J*_2_ but not for *J*_1_, which varies between −0.2 meV in VASP and −7.2 meV in FPLO [35]. The authors pointed out that this difference comes from the way the DFT+U correction is applied in different codes.

In [12], the NN and NNN exchange interactions within the chain are substantially larger compared to the values obtained previously [11,22] and shift the frustration ratio *α* ≈ 0.32 of PbCu(SO_4_)(OH)_2_ closer to the one-dimensional critical point at 0.25. On the contrary, the present calculation of the main intrachain exchange interaction parameters put PbCu(SeO_4_)(OH)_2_ very close to the Majumdar–Gosh critical point. Similar to the case of CuCrO_4_, we do not observe the formation of the singlet ground state, but presumably the incommensurate helix forms in the magnetically ordered state of this compound. Similar to the mineral linarite case, there were multiple indicators of the formation of an additional electrically active phase preceding the long-range magnetic order, which suggests that PbCu(SeO_4_)(OH)_2_ is a multiferroic system.

## 6. Conclusions

A selenium analog of the naturally occurring mineral linarite was prepared hydrothermally for the first time. This quasi-one-dimensional compound evidences formation of a short-range correlation regime at T* ~ 8 K and experiences a long-range magnetic order at *T_N_* = 4.3 K. A magnetization saturation of approximately 1 *µ_B_* is reached at *µ_0_H_flip_* ~ 16 T preceded by a jump at *µ_0_H_flop_* = 2.4 T. Additionally, there are multiple indicators of the formation of an additional electrically active phase above the Neel temperature, which suggest that PbCu(SeO_4_)(OH)_2_ is a multiferroic system. It should be noted that during manuscript preparation, the mineral franksousaite, which has a similar composition, was described [38]. To the best of our knowledge, there are no synthesis descriptions for the linarite or franksousaite compound. The crystal structure of synthetic PbCu(SeO_4_)(OH)_2_ is close to that of franksousaite, taking into account the presence of approximately 16% of sulfur in the natural sample.

## Figures and Tables

**Table 1:** Selected crystallographic data and refinement parameters for PbCu(SeO_4_)(OH)_2_ from single-crystal X-ray diffraction.

Chemical Formula	CuH_2_O_6_PbSe
Formula mass [g mol^–1^]	447.71
Crystal size, mm	0.120 × 0.040 × 0.030
Crystal system	Monoclinic
Space group	*P2*_1_/*m* (no. 11)
*a* [Å]	4.748(2)
*b* [Å]	5.745(3)
*c* [Å]	9.822(9)
*β*	102.39(3)
*V* [Å^3^]	261.7(3)
*Z*	2
*T* [K]	110(2)
*d*_calcd._ [g cm^3^]	5.681
*μ* [mm^–1^]	43.091
*θ* range [°]	2.065 < *θ* < 29.994
Collected/independent reflections	2740/829
*R*_σ_/*R*_int_	0.0353/0.0329
Reflections with *I* > 2*σ*(*I*)	789
Refined parameters	52
Largest difference peak/hole [e Å^–3^]	1.632/−1.352
*R*_1_ [*I* > 2*σ*(*I*)]/*R*_1_ [all data]	0.0259/0.0277
*wR*_2_ [*I* > 2*σ*(*I*)]/*wR*_2_ [all data]	0.0614/0.0614
*GoF*	1.075

**Table 2:** Selected bond distances in the PbCu(SeO_4_)(OH)_2_ crystal structure.

Bond		Distance, Å
Pb—O5		2.346(12)
Pb—O2	x2	2.413(20)
Pb—O3	x2	2.832(35)
Pb—O1	x2	3.029(2)
Se—O3		1.637(26)
Se—O1		1.641(6)
Se—O2	x2	1.654(6)
Cu—O4	x2	1.930(8)
Cu—O5	x2	1.994(17)
Cu—O2	x2	2.483(9)

**Table 3:** The exchange interaction parameters (in K) calculated within GGA+U for PbCu(SeO_4_)(OH)_2_ and PbCu(SO_4_)(OH)_2_. The notations *J*_1*c*_, *J*_2*c*_ and *J*_3*c*_ correspond to the NN, NNN and NNNN interchain exchange interactions [12].

Compound	U, eV	*J*_1_, *K*	*J*_2_, *K*	*J*_1*c*_, *K*	*J*_2*c*_, *K*	*J*_3*c*_, *K*	*α* = |*J*_2_/*J*_1_|
PbCuSO_4_(OH)_2_	7	−118	72	13	−3	1	0.61
PbCuSeO_4_(OH)_2_	7	−103	54	6	−1.5	1	0.52
PbCuSeO_4_(OH)_2_	9	−80	33	4	−1	0.6	0.41
